# A practical guide for choosing an optimal spatial transcriptomics technology from seven major commercially available options

**DOI:** 10.1186/s12864-025-11235-3

**Published:** 2025-01-20

**Authors:** Hyun Ju Lim, Ye Wang, Anton Buzdin, Xinmin Li

**Affiliations:** 1 UCLA Technology Center for Genomics & Bioinformatics, Department of Pathology & Laboratory Medicine, 650 Charles E Young Dr. South, Los Angeles, CA 90095 USA; 2 Clinical Laboratory, The Affiliated Qingdao Central Hospital of Medical College of Qingdao University, Qingdao, 266042 China; 3 World-Class Research Center “Digital biodesign and personalized healthcare”, I.M. Sechenov First Moscow State Medical University, Moscow, 119991 Russia; 4 Shemyakin-Ovchinnikov Institute of Bioorganic Chemistry, Moscow, 117997 Russia

**Keywords:** Spatial transcriptomics, RNA sequencing, 10X Visium, 10X visium HD, GeoMx DSP, CosMx SMI, Merscope, Stereoseq, Xenium

## Abstract

Spatial transcriptomics technology enables the mapping of gene expression within tissues, allowing researchers to visualize the spatial distribution of RNA molecules and gain insights into cellular organization, interactions, and functions in their native environments. A variety of spatial technologies are now commercially available, each offering distinct technical parameters such as cellular resolution, detection sensitivity, gene coverage, and throughput. This wide range of options can make it challenges or create confusion for researchers to select the most appropriate platform for their specific research objectives. In this paper, we will analyze and compare seven major commercially available spatial platforms to guide researchers in choosing the most suitable option for their needs.

## Background

Cells, the fundamental units of life, are elaborately organized to form diverse tissues and organs. This sophisticated organization of cells in space defines the structure of living organisms and their specific functions. Genes encode proteins that undertake various tasks within the cell, ultimately governing its structure, function, and behavior. Therefore, comprehending the precise locations where genes are expressed within highly structured tissues is essential for elucidating various fundamental biological processes, such as gene functions, gene-gene interactions, cell-cell communication, dynamic molecular and cellular processes, and microenvironmental influences [1].

However, this functionally critical spatial information is lost in widely used bulking sequencing and popular single cell sequencing due to the disruption of tissue structural organization during the sample process. Given this critical weakness of bulk and single cell sequencing, and driven by recent technological advances, the landscape of spatial technologies is flourishing at an unprecedented pace in the past few years (For recent review, see You Y et al. [2]; Ren JY et al. [3]; Gulati GS et al. [4]; Jain S and Eadon M [5]). Now, there are at least 9 spatial technologies commercially available, each with unique strengths and inherent weaknesses. In this article, we focus on seven widely used spatial platforms: 10X Visium [6], GeoMx DSP (GeoMx) [7], Visium HD [8], Stereoseq [9], Merscope [10], Xenium [11] and CosMx SMI (CosMx) [12]. We begin by highlighting the core technologies of these spatial platforms, offering a foundation for understanding their technological differences. This is followed by a comparative analysis of their key technical features to help researchers evaluate their capabilities. Finally, we provide guidance on key factors, such as experimental aims, available resources, and specific requirements, for selecting the most suitable spatial technology to address particular biological questions.

## Technology outline

Spatial transcriptomics can be broadly categorized into two groups: imaging-based and sequencing-based technologies [13, 14]. While both approaches reveal the spatial locations of gene expression, their underlying technologies differ drastically in capturing spatial information and determining abundance of specific mRNA molecules within tissue.

### Imaging-based technologies

**Fig. 1:**
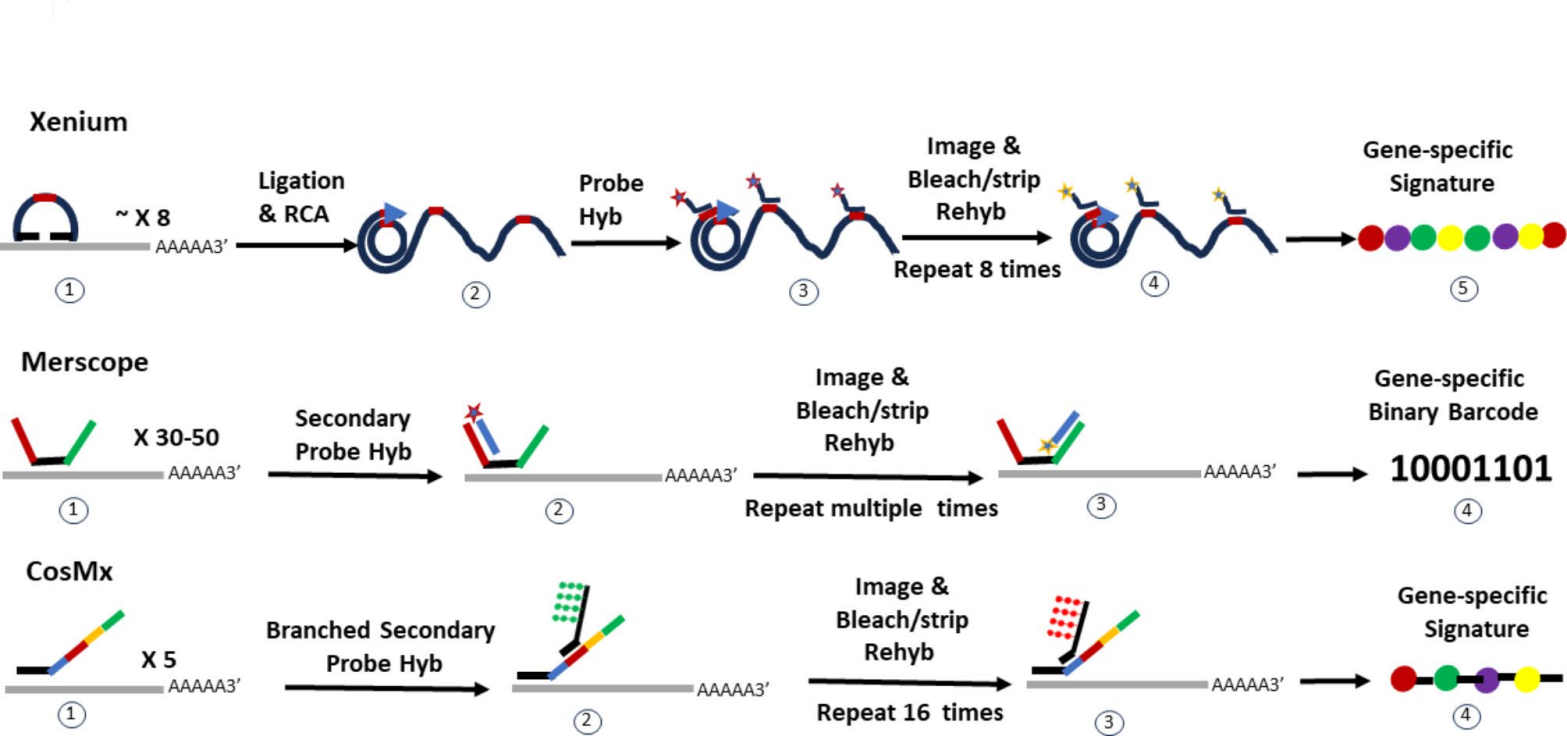
Illustration of Different Probe Design, Probe Hybridization, Signal Amplification and Gene Decoding Methods for Xenium, Merscope, and CosMx. **Xenium**: (**1**) 8 padlock probes hybridize to targeted mRNA, (**2**) padlock probe ligation, followed by rolling circle amplification (RCA), (**3**) fluorescently labeled secondary probes hybridize to the padlock probes, (**4**) imaging the fluorescent signal, followed by fluorophore removal and next rounds of hybridization with different fluorophores, these processes repeat 8 times, (5) optical gene-specific signature generation; **Merscope**: (**1**) 30–50 gene-specific primary probes hybridize to targeted mRNA, (**2**) fluorescently labeled or unlabeled secondary probes hybridize to one of the primary probe tails, (**3**) imaging the fluorescent signal (detection of fluorescence give a “1” and the absence of fluorescence give a “0” in the barcode), signal stripping and new round of secondary probe hybridization, (**4**) gene-specific binary barcode generation; **CosMx**: (**1**) 5 gene-specific primary probes hybridize to targeted mRNA, (**2**) fluorescently labeled, branched secondary probes attaching multiple fluorophores hybridize to one of the 16 sub-domains, (**3**) imaging the fluorescent signal, followed by fluorophore removal and next rounds of hybridization with different fluorophores. This cycle repeats 16 times, (**4**) positional optical gene-specific signature generation.

Imaging-based technologies employ single-molecule fluorescence in situ hybridization (smFISH) [15] as their backbone technology. These technologies enable the simultaneous detection of up to six thousand RNA transcripts in a single experiment through cyclic, highly multiplexed smFISH. This is achieved by using dozens of primary probes that hybridize to specific RNA transcripts, followed by secondary probes labeled with different fluorophores hybridizing to the primary probes. By sequentially hybridizing and imaging fluorescence from these secondary probes, researchers can determine the spatial location and expression levels of individual RNA transcripts within tissues based on transcript-specific fluorescent signatures and their intensity. The differences between imaging-based platforms are mainly in probe design, probe hybridization, signal amplification and gene decoding (Fig. 1). We briefly outline the key technologies of Xenium, Merscope and CosMx below.

#### Xenium

Xenium is a hybrid technology combining in situ sequencing (ISS) and in situ hybridization (ISH) in two main steps. (1) Probe Hybridization and Amplification: An average of 8 padlock probes, each containing a gene-specific barcode, hybridize to the target RNA transcript. Upon successful binding, these probes undergo highly specific ligation to form circular DNA constructs, which are then enzymatically amplified through rolling circle amplification (RCA), producing multiple copies of the circular DNA to enhance signal sensitivity. (2) Signal Detection and Imaging: fluorescently labeled oligonucleotide probes bind to the gene-specific barcode within the padlock probe. After imaging the fluorescent signal, the fluorophores are removed, allowing successive rounds of hybridization with different fluorophores. This process is repeated on average 8 times, using a distinct fluorophore in each round to generate a unique optical signature that corresponds to the identity of the target gene. This padlock design, along with its subsequent amplification, enables accurate, sensitive, and specific detection and spatial localization of gene activity within tissue samples [16].

#### Merscope

Unlike the optical signature method described above, Merscope technology utilizes a binary barcode strategy for gene identification. Each gene is assigned a unique binary barcode, consisting of a series sequence of “0"s and “1"s. (1) Probe Hybridization: Thirty to fifty gene-specific primary probes hybridize to different regions of the targeted gene. Each primary probe has a target-binding domain for RNA hybridization and “hangout tails” for secondary probe binding. (2) Barcode Decoding: Fluorescently labeled or unlabeled secondary probes bind to these tails to read the barcode. The decoding process occurs over multiple rounds of imaging, signal stripping and new secondary probe introduction. During each round, the detection of fluorescence corresponds to a “1” in the barcode, while the absence of fluorescence corresponds to a “0”. Typically, a Merscope barcode contains four “1"s in a predetermined order, meaning the fluorescent signal for any given gene is detected only four times across the imaging rounds. This process generates a barcode that is then matched to the pre-assigned binary barcode to identify and quantify the transcript. This binary barcoding strategy reduces optical crowding and supports error correction in readouts [10].

#### CosMx

CosMx employs a hybridization method similar to MERSCOPE and an optical signature approach like Xenium, while incoporating an additional positional dimension for gene identification. (1) Primary hybridization: The process begins with a pool of five gene-specific probes, each containing a 30–50 nucleotide target-binding domain and a 100-nucleotide readout domain. The readout domain consists of 16 sub-domains, each of which bind to fluorescently labeled secondary probes. Within each pool, the probes have unique sequences in the target-binding domain but share the same sequence in the readout domain. (2) Secondary probe binding and signal amplification: Each secondary probe includes a primary probe binding domain linked to a branched, fluorescently labeled readout domain through a UV-cleavable linker. The branched readout domain allows attaching multiple fluorophores to effectively enhance signal intensity. (3) Imaging and Cyclic Readout: After imaging, UV light cleaves the fluorescent labeling domain, enabling a new round of secondary probe hybridization. This cycle repeats 16 times. (4) Gene Identification: The four fluorescent colors and 16 sub-domains generate a unique combination of color and position signature for each target gene. This combinational readout strategy enables CosMx to identify and quantify more target genes than Xenium and MERSCOPE [12].

### Sequencing-based technologies

**Fig. 2:**
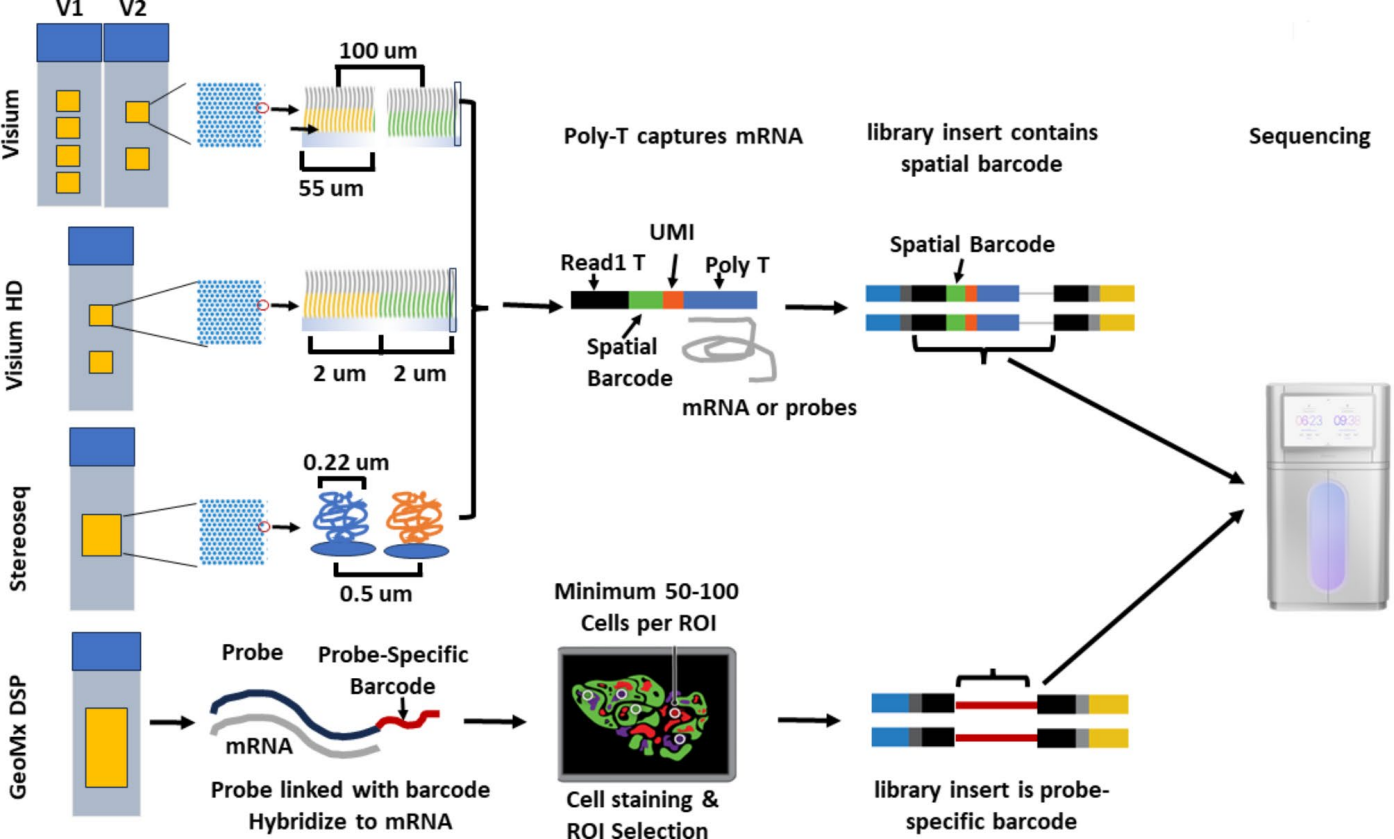
Depiction of Different Feature/ROI Size and Decoding Spatial Information Method for Visium, Visum HD, Stereoseq and GeoMx. The fundamental difference among Visium, Visium HD, and Stereo-seq lies in the feature size of their arrays: 55 μm for Visium, 2 μm for Visium HD, and 0.22 μm for Stereo-seq. A shared characteristic of these three technologies is their ability to capture mRNA (Visium V1 and Stereo-seq for fresh tissues) or probes (Visium HD) via Poly-T oligonucleotides on the array for downstream library construction and sequencing (as illustrated in the figure). However, Stereo-seq’s FFPE workflow employs a ligation-mediated approach to capture random primer probes. This detail is not shown in the figure, as the specific methodology has not been published as of November 28, 2024. In addition, Stereoseq libraries need to be sequenced on a DNBSEQ-T7 sequencer. During library construction, spatial barcodes are incorporated into the libraries to enable spatial mapping in subsequent analyses. In contrast, GeoMx employs a pool of gene-specific probes, each linked to a unique DSP barcode via a UV-cleavable linker, to hybridize with mRNA targets. After cell staining, regions of interest (ROIs) are selected, and the DSP barcodes are released from the gene-specific probes through UV exposure. These barcodes are then collected for downstream library construction and sequencing. The sequenced DSP barcodes enable targeted mRNA identification and quantification, while the ROIs allow spatial mapping of the transcripts.

Unlike imaging-based technologies, most sequencing-based technologies, including 10X Visium, 10X Visium HD and Stereoseq, integrate spatially barcoded arrays with next-generation sequencing to determine the locations and expression levels of transcripts within tissues (Fig. 2). In most cases, this is achieved by capturing mRNA transcripts within the tissue using a polyT tail built into the unique, spatially barcoded probes on the array. During cDNA synthesis, these spatial barcodes are incorporated into each cDNA molecule. By subsequent library construction and sequencing, researchers can determine the expression levels of transcripts and map them back to their precise locations within the tissue based on their spatial barcodes. The fundamental difference among these technologies is the feature size of the array, which largely determines spatial resolution. Although the GeoMx is considered a sequencing-based technology, it employs a combinational strategy of barcoded probes and region-of-interest (ROI) selection to determine spatial transcript location. The transcript-specific barcodes are then sequenced to uncover the identity and quantity of each transcript. Below, we outline the key differences among these sequencing-based technologies.

#### 10X Visium and Visium HD

The core technology of Visium relies on spatially barcoded RNA-binding probes attached to the Visium slide. These probes contain several domains, including spatial barcode for decoding the spatial location of the mRNA, a random molecular tag (UMI) for identifying and quantifying unique mRNA transcript, and an oligo-dT sequence for mRNA binding. Visium technology offers two versions of workflows: V1 for fresh tissue, and V2 for both fresh and FFPE tissue with a modified mRNA capture strategy [17]. The V2 workflow requires a CytAssist instrument, a compact instrument designed to simplify the process by transferring gene-specific transcriptomic probes from standard glass slides onto the Visium slide for improved sample handling. (1) V1 Workflow: After tissue permeabilization, released mRNA binds directly to the poly(dT) region of adjacent RNA-binding probes on the Visium slide. Double-stranded cDNA is then synthesized on-slide, with the second-strand cDNA—carrying the spatial barcode—collected off-slide for library preparation and sequencing. (2) V2 Workflow: In this version, a pair of adjacent probes hybridizes to target mRNA, and the probe pair is then ligated to form a longer probe. The poly-A tail linked to one of the probes at the 3’ end is captured by the poly(dT) on the Visium slide (this probe hybridization approach in V2 is optimized for handling degraded RNA, making it suitable for FFPE samples). The final library is made following probe releasing, probe extension to incorporates spatial barcode, pre-amplification and final library amplification. Visium HD uses the same technology as the Visium V2 workflow but features a significantly smaller spot size of 2 μm, compared to the standard 55 μm feature size in Visium. This smaller feature size enhances spatial resolution (see the next section for a detailed technology comparison).

#### Stereoseq

Stereo-seq utilizes DNA nanoball (DNB) technology for in situ RNA capture. Briefly, the synthesized oligo probes contain multiple domains, including random barcoded sequences, coordinate identity (CID), molecular identifiers (MID), and a poly(dT) sequence (in case of fresh tissue workflow). Unlike 10X Visium, which directly attaches oligo probes to the slide, Stereo-seq oligo probes are circularized and used as templates to generate DNA nanoballs (DNBs) via rolling circle amplification (RCA). The DNBs are then loaded onto a grid-patterned array to create the Stereo-seq capture slides. With a diameter of approximately 0.2 μm and a center-to-center distance of 0.5 μm, the DNBs are significantly smaller than the 2 μm spots in Visium HD [18]. The spatial position of each DNB is then identified by sequencing the 25-nucleotide CID. Stereoseq adopted different hybridization strategies for fresh tissue and FFPE tissue: (1) Fresh Tissue Workflow: Similar to Visium V1, Stereoseq use poly-dT sequences embedded in oligo probes to capture mRNA from fresh tissue. (2) FFPE Tissue Workflow: Unlike the Visium V2 FFPE workflow, which uses gene-specific probes to hybridize with target RNA, Stereoseq employs random primers to capture RNA from permeabilized FFPE tissue sections. The captured RNAs are then reverse-transcribed and ligated onto the Stereoseq chip for library construction and sequencing [19].

#### GeoMx

Different from other sequencing-based technologies, GeoMx utilizes NanoString’s digital molecular barcoding technology to identify the spatial distribution of transcripts within tissue. (1) Probe Hybridization: A pool of gene-specific probes is designed for protein-coding genes, with each probe linked to a unique digital spatial profiling (DSP) barcode via a UV-cleavable linker. This barcoded probe pool is hybridized to mRNA targets within tissue sections mounted on glass slides. (2) Regions of interest (ROI) Selection: Fluorescent markers are then applied to stain cells of interest, enabling the selection of ROIs based on the stained cells. (3) Barcode Collection: When the tissue is exposed to UV light, the DSP barcodes are released from the probes within the ROIs and collected for downstream processing [7]. (4) Library Construction, Sequencing, and Spatial Mapping: The released DSP barcodes are used for library construction and sequencing. Each unique DSP barcode corresponds to a specific gene, and the barcode counts reflect mRNA abundance. The spatial location of each mRNA is determined by mapping the DSP barcodes back to the tissue section in the defined ROIs.

In general, imaging-based technologies offer single-cell or subcellular resolution, high RNA detection sensitivity, specificity, and reproducibility. However, they are limited to gene panels ranging from several hundred to six thousand genes, require longer imaging times varying from two days to a week or more, and have low throughput. Sequencing-based technologies provide whole transcriptome analysis, though most lack single-cell resolution. They typically have lower RNA capture efficiency and detection sensitivity compared to imaging-based technologies, especially for low-abundance transcripts [20]. These limitations can be improved by increasing sequencing depth.

## Technology comparisons

To facilitate informative comparisons across various technologies, we categorize the seven spatial platforms into three groups based on spatial resolution and key characteristics. We then compare their main features and capabilities within each group. For quick reference, a summary of each platform is provided in Table 1.

### Group 1 (G1)

**10X Visium and GeoMx**. G1 platforms share many common features although they employ different technologies to define spatial content. Both are sequencing-based, compatible with both fresh and FFPE samples, capable of performing whole transcriptome and protein panel analyses, but both are lack of single cell resolution. The key differences between these two platforms are summarized in Table 1. It is worth noting that GeoMx is limited to human and mouse samples only, whereas 10X Visium V1 can work with any species using fresh tissues. However, when CytAssist is utilized with V2 kit, 10X Visium also becomes restricted to human and mouse samples. Given the large imaging area and the ability to select ROIs, GeoMx is particularly suitable for analyzing many small-sized samples, such as biopsies, or a single large tissue section with scattered cells of interest. One prerequisite for choosing GeoMx is that users must identify the cells of interest beforehand, as these cells will be stained during the ROI selection process. If specific cells of interest are not predefined or unknown, 10X Visium is more suitable for unbiased discovery. In general, Visium offers superior spatial resolution and specificity, while GeoMx is more flexible with multiple samples/slide.

The key strength of this group is its ability to deliver robust whole-transcriptome analysis data. The main limitation is the lack of single-cell resolution, which is due to the ≥ 55 μm spot size in Visium (the average size of a mammalian cell is typically around 10 μm in diameter) and the ≥ 50-cell ROI size (Vendor recommended) in GeoMx. Consequently, each spot or ROI captures transcripts from multiple cell types, resulting in an average or mixed gene expression signal across these cell types. To address this limitation, deconvolution tools have been developed and commonly used in analyzing G1 data. By integrating spatial transcriptomic data with single-cell RNA sequencing data from adjacent tissue sections, deconvolution can assign specific gene expression profiles to distinct cell types within each spot, allowing for a more accurate understanding of cellular architecture in tissues [21, 22].

### Group 2 (G2)

**Visium HD and Stereoseq.** Group 2 platforms share the same sequencing-based technologies as G1 platforms, but with substantially smaller feature size, resulting in significantly higher resolution. G2 platforms are compatible with either fresh tissue or FFPE tissue. As of November 28, 2024, G2 group is limited to transcriptome analysis only. A key distinction between Visium HD and Stereoseq is their compatibility with different species (Table 1). Visium HD utilizes a probe hybridization method to capture mRNA from both fresh and FFPE tissue for human and mouse samples. In contrast, Stereoseq employs a poly-A approach for mRNA capture in fresh tissues and random primers for FFPE tissue, enabling transcriptome analysis across all species with either fresh or FFPE tissues. Another obvious difference is the size of their capturing area. Visium HD has a 6.5 mm x 6.5 mm capture area, while Stereoseq offers larger options, including 10 mm x 10 mm and 20 mm x 30 mm capture area to accommodate bigger tissue sizes. Visium HD features a 2 μm spot size without gap between spots, providing single-cell scale resolution, whereas Stereo-seq has a feature size that is 10 times smaller (0.2 μm), allowing data analysis at a single cell resolution. However, it is important to note that feature size is not the only factor influencing actual resolution; lateral diffusion during tissue permeabilization also significantly impacts the spatial accuracy of transcript detection. Stereoseq has shown notable lateral diffusion in some tissues [2]. In this context, optimizing permeabilization time for different tissue types is crucial to minimizing diffusion and preserving resolution. Additionally, the term ‘single-cell resolution’ can be somewhat misleading. Platforms like Visium HD and Stereo-seq can detect only a few hundred to a thousand genes when data are exported to achieve single-cell scale/resolution. This level of resolution comes at the expense of a reduced number of detectable genes. As of now, sequencing-based spatial technologies are not yet capable of delivering whole-transcriptome data at true single-cell resolution.

### Group 3 (G3)

**Merscope, Xenium, and CosMx.** The common features of G3 platforms are imaging-based, compatible with both fresh frozen and FFPE tissues, and able to profile a variable number of genes at sub-cellular resolution. However, there are several technical differences across the three platforms as summarized in Table 1. While all three platforms provide pre-designed panels for human and mouse, Merscope distinguishes itself as an open platform, offering full customization of up to a 1000-gene panel for any species without any design fees. In contrast, Xenium and CosMx technically support custom gene panel creation for any species, the associated high design fees make it less practical to opt for a fully custom-designed panel. However, these platforms allow users to add up to 200 custom genes to an existing gene panel to address certain specific needs. Another distinctive feature of Merscope is its capability to profile cultured cells, enabling the examination of spatial intracellular signaling in complex cell cultures such as 3D structures or organoids. Merscope enables simultaneous RNA and protein profiling on the same slide, while CosMx performs this sequentially, and Xenium does not currently support protein panel. Both Xenium and Merscope demonstrate superior specificity compared to CosMx [23]. Xenium exhibits exceptional sensitivity with FFPE tissues, while Merscope outperforms Xenium with high-quality tissue [24].

**Table 1:** Comparative summary of key features and capabilities across different spatial platform

Key Features	Sequencing-Based Platforms				Imaging-Based Platforms		
	Group 1		Group 2		Group 3		
	10X Visium	GeoMx DSP	10X Visium HD	Stereoseq	Merscope	Xenium	CosMx SMI
**Suitable species**	V1: Any species V2: Human and mouse only	Human & mouse only	Human & mouse only	Any species	Any species	Human & mouse only	Human & mouse only
**Applicable tissue types**	*FF, FFPE	FF, FFPE	FF, FFPE, **FxF	FF, FFPE, **FxF	FF, FFPE and cultured cells	FF, FFPE	FF, FFPE
**Capture (Imaging) area/Slide**	V1 Kit: Four 6.5 mm x 6.5 mm V2 Kit: Two 6.5 mm x 6.5 mm	36 mm x 14 mm	Two 6.5 mm x 6.5 mm	One 10 mm x10mm or one 20 mm x 30 mm	18 mm X 22 mm	10 mm X 22 mm	15 mm X 20 mm
**Number of genes profiled**	Whole transcriptome	Whole transcriptome	Whole transcriptome	Whole transcriptome	Predesigned panels: 500 genes + ~ 500 custom genes Custom-designed panels: up to 1000	Predesigned panels: up to 5000 genes + ~ 100 custom genes.	Predesigned panels: up to 6000 + ~ 200 custom genes
**Number of proteins profiled**	35- sequential detection of protein and RNA on separate slides	570- sequential detection of protein and RNA on separate slides	Not available now	Not available now	6 - simultaneous co-imaging of protein and RNA on the same slide	Not available now	68 -sequential detection of protein and RNA on separate slides
**Resolution**	55 μm	> 55 μm ( > = 50 cells for each ROI)	Single Cell scale (2 μm feature size)	Single Cell (0.22 μm feature size)	Single Cell/Subcellular	Single Cell/Subcellular	Single Cell/Subcellular
**Sequencing/** **Capture area**	V1 Kit: 250 M reads (*FF); V2 Kit: 125 M reads (FFPE)	Variable dependent on ROI size	FFPE: 275 M reads; *FF: 700 M reads; **FxF: 500 M reads	*FF: 1.5B reads FFPE: 3B reads (10mmx10mm)	Not applicable	Not applicable	Not applicable
**Hands-on time**	1–2 days	3 days	2 days	2–3 days	4–5 days	3 days	2 days
**Scanning time**	Not applicable	Not applicable	Not applicable	Not applicable	1–2 days	2–6 days	3-7days
**Specificity** ^**#**^	High	Low	Not Available	Not Available	High	High	Low
**Cost** ^**&**^	$3293 + seq	$3837 + seq	$6645 + seq	$3054 + seq	~$6733	$3878	$6325
**Experimental aim**	Discovery purpose	Focused study with known cells of interest	Discovery purpose & Precision Insight	Discovery purpose & Precision Insight	Precision Insight	Precision Insight	Precision Insight

*FF: Fresh Frozen; **FxF: Fixed Frozen; # The comparisons are made Only within each group. ^&^Estimated based on current rates at UCLA Technology Center for Genomics & Bioinformatics. The estimates will change with different panels and over time.

## Experimental considerations/requirements

After becoming familiar with the technical capabilities of different spatial technologies, the next step is to define your biological questions, current resources, and experimental needs to determine the best approach.

### Defining your biological questions

Given the high cost of spatial experiments, it is essential to clearly define your biological questions to ensure that spatial technology is the appropriate approach. In other words, the spatial content in the dataset is crucial for answering your questions that cannot be satisfactorily addressed by other simpler or more cost-effective technologies. Examples of such questions include understanding how tumor cells interact with immune cells, how the tumor microenvironment affects tumor metastasis, and how drug treatment activates immune pathways, facilitates immune cell infiltration, and impacts tumor cell viability. If your aim is to address these dynamic interaction processes in situ, spatial context is essential.

### Understanding your resources

During the experimental planning stage, it is important to have a thorough understanding of the sample details, including species, tissue type and size, cell of interest, RNA integrity and your budget. This information will guide the selection of the most appropriate platform.

#### Species

If you are working with non-human and -mouse samples, your options are practically limited to Stereoseq and Visium V1 kit for sequencing-based platforms, and Merscope for imaging-based platforms. Other platforms typically limit pre-designed panels to human and mouse models only. As mentioned earlier, the cost of designing custom panels for these platforms is often prohibitive.

#### Tissue size

Given the different platforms have very different capturing areas, the size of the tissue is an important factor to consider when selecting a platform. If you plan to use Visium or Visium HD, the tissue must fit within the 6.5 mm x 6.5 mm capture area. In contrast, other platforms offer larger capture area of at least 1 cm x 1 cm, providing more flexibility for tissue size.

#### Cells of interest in the tissue

If you are interested in specific cell type(s), it is essential to understand their distribution within the tissue sample. Are these cells evenly distributed, or do they appear in small, scattered clusters? GeoMx, with its capability to select specific ROIs within the tissue, is particularly effective for analyzing clustered distributions. In such cases, other sequencing-based platforms may lead to higher sequencing costs without providing additional benefit. The ability to select ROIs and large capture area makes GeoMx a preferred choice for analyzing larger tissues with clustered cells.

#### RNA integrity

Different spatial platforms employ various RNA capture and probe hybridization strategies, each with distinct capabilities to address varying levels of RNA quality. For instance, Xenium has better ability to handle degraded RNA, while Merscope outperforms Xenium with high-quality RNA [18]. Generally, a RIN of ≥ 7 is recommended for fresh tissue, and a DV200 of ≥ 30% is needed for FFPE tissue.

#### Budget

Costs vary significantly across platforms. For example, Visium HD is twice as expensive as Stereo-seq for library construction, despite analyzing fewer cells. Xenium is the most affordable platform among imaging-based options. When evaluating costs, keep in mind that imaging-based platforms have no additional sequencing costs but analyze fewer genes and cover more cells. Drafting a rough budget can help ensure your platform choice aligns with both your financial resources and project goals.

### Identifying your specific needs

After assessing your current resources, the next step is to define your specific requirements, including.

#### Number of genes profiled

Decide whether you need whole transcriptome analysis for unbiased discovery or targeted analysis for more precise insights. This choice should align with your experimental objectives. Typically, if the goal is to generate hypotheses, sequencing-based platforms like 10X Visium and GeoMx are ideal, as they provide an unbiased view of the whole transcriptome. If the aim is to test hypotheses, imaging-based platforms such as Xenium, CosMx, and Merscope are optimal, offering in-depth analysis of a specific gene panel at single-cell resolution. For those seeking flexible control over both resolution and the number of genes analyzed, Visium HD or Stereo-seq are better choices. By adjusting the bin size of the data output, you can balance resolution with gene coverage: lower resolution supports whole transcriptome analysis, while higher resolution detects fewer genes.

#### Analyte

Decide whether you’re interested in profiling RNA alone or both RNA and protein. As of November 28, 2024, Visium HD, Stereoseq, and Xenium are limited to transcriptomic (RNA) analysis only. The remaining platforms are capable of both RNA and protein profiling.

#### Spatial resolution

Consider whether you need single-cell resolution to visualize analytes within individual cells. Imaging-based platforms offer single-cell and subcellular resolution, while Visium HD and Stereo-seq provide options to analyze data at single-cell scale. In contrast, Visium and GeoMx have a spatial resolution of 55 μm or larger.

#### RNA capturing efficiency

Imaging-based platforms generally have higher RNA capture efficiency compared to sequencing-based platforms. Among imaging-based platforms, Xenium provides better capturing efficiency than CosMx and Merscope.

#### Imaging/capturing area

Platforms like 10X Visium V2 and Visium HD have two capture areas of 6.5 mm x 6.5 mm per slide (four areas with Visium V1), while others provide larger capture area of ≥ 10 mm x 10 mm per slide.

#### Sensitivity & specificity

Different platforms vary in sensitivity and specificity. Imaging-based platforms generally offer superior detection sensitivity and specificity compared to sequencing-based platforms. Among sequencing technologies, probe-based methods outperform polyA-based approaches, particularly for detecting lowly expressed genes. Studies indicate that 10X Xenium and Merscope technologies provide better sensitivity and specificity than CosMx [23, 24]. Probe-based Visium technology is considered more effective than Stereo-seq [2].

Based on your experimental goal, available resources, specific requirements, and key features of various spatial platforms, you can make an informed decision to select the most suitable platform for your experimental objectives. To assist with your decision making, Table 2 presents a simplified decision guide. This table covers key parameters for easy reference but is not exhaustive. Spatial transcriptomics technologies are still in the early stages of development and are rapidly evolving. The performance of each technology is likely to improve over time with ongoing advancements. Therefore, this practical guide will need to be updated accordingly.

**Table 2:** Simplified decision guide for selecting the optimal platform

Key Parameters to Consider		10x Visium V.1	10x Visium V.2	GeoMx	10x Visium HD	Stereoseq	Merscope	Xenium	CosMx
**Species**	**Human & Mouse Only**		✓	✓	✓			✓	✓
	**All**	✓				✓	✓		
**Experimental Aim**	**Hypothesis Generation**	✓	✓	✓					
	**Hypothesis Generation & Testing**				✓	✓			
	**Hypothesis Testing**						✓	✓	✓
**Resolution**	**>= 50 μm**	✓	✓	✓					
	**Single Cell Scale/Single Cell**				✓	✓			
	**Single Cell/Subcellular**						✓	✓	✓
**Gene Profiled**	**WTA**	✓	✓	✓	✓	✓			
	**Gene Panel**						✓	✓	✓
**Protein Profiled**	**Protein Panel**	✓	✓	✓			✓		✓
**Tissue Size**	**≤ 6.5 mm X 6.5 mm**	✓	✓		✓				
	**> 6.5 mm X 6.5 mm**			✓		✓	✓	✓	✓
**Cost in relative to cells analyzed**	**Low**					✓		✓	
	**Medium**	✓	✓	✓					
	**High**				✓		✓		✓

## Move forward

Spatial technologies are advancing rapidly, with key areas for further improvement, such as enhancing spatial resolution without significantly reducing the number of detectable genes for sequencing-based technologies and increasing the number of profiled genes without substantially extending scanning time for imaging-based technologies. As progress is made in overcoming these challenges, along with the development of new bioinformatics tools and algorithms, spatial technologies are poised to become a dominant tool in the coming years, but they have not yet reached its full potential.

One future direction for spatial genomics is the development of 3D spatial multiomics technologies. StellarOmics’ imaging-based in-situ sequencing technology shows great promise in this area [25]. Unlike the standard 5–10 μm thick sections used in current spatial technologies, StellarOmics can image tissue sections up to 200 μm thick, enabling multi-cell layer profiling for deeper insights into tissue architecture and function. We anticipate continued advancements in this direction in 2025 and beyond.

## Data Availability

No datasets were generated or analysed during the current study.
